# P72 Investigating the adaptive evolutionary responses of MRSA to alkylating agents

**DOI:** 10.1093/jacamr/dlaf230.079

**Published:** 2025-12-04

**Authors:** Youssef Hamdy, Hossam B El-Geneidy, Mohamed Elhadidy

**Affiliations:** Center for Genomics, Helmy Institute for Medical Sciences, Zewail City of Science and Technology, Giza, Egypt; Biomedical Sciences Program, University of Science and Technology, Zewail City of Science and Technology, Giza, Egypt; Center for Genomics, Helmy Institute for Medical Sciences, Zewail City of Science and Technology, Giza, Egypt; Biomedical Sciences Program, University of Science and Technology, Zewail City of Science and Technology, Giza, Egypt; Center for Genomics, Helmy Institute for Medical Sciences, Zewail City of Science and Technology, Giza, Egypt; Biomedical Sciences Program, University of Science and Technology, Zewail City of Science and Technology, Giza, Egypt; Department of Bacteriology, Mycology and Immunology, Faculty of Veterinary Medicine, Mansoura University, Mansoura, Egypt

## Abstract

**Background:**

Disinfectants are antimicrobial agents frequently used in healthcare settings. Bacterial susceptibility against a wide range of antimicrobial agents can be changed due to prolonged exposure to sub-inhibitory concentrations of disinfectants.

**Objectives:**

To investigate the adaptive responses of hospital-acquired MRSA (HA-MRSA) to long-term exposure to alkylating agents such as Glutaraldehyde (GA) and Formaldehyde (FA). In a 25 day serial passaging, isolates were exposed to different inhibitory concentrations. Bacterial isolates were selected based on genetic diversity across the clonal complexes (CCs) and antibiotic resistance profiles. MICs were conducted before and after passaging for all isolates, indicating consistent MIC values for FA and GA in the WT strain.

**Results:**

After 25 days, three different strains evolved in GA showed shifting in the MIC values, indicating lower susceptibility for GA. In addition to the MIC analysis, efflux pump activity and biofilm formation capabilities were evaluated for all isolates prior to and post the long-term exposure. Different efflux pump activities were observed with WT samples; however, evolved isolates, belonging to CC22, showed higher efflux activity in response to GA and FA. Additionally, isolates were classified based on their biofilm formation abilities. WT strains exhibited a wide range of forming abilities. Among evolved isolates, all screened ones were prone to changes in their formation abilities; however, the highest changes were more related to FA.

**Conclusions:**

These findings highlight that evolutionary responses are difficult to predict, as they vary depending on multiple factors such as the type of stressor, the CC of the isolate, and its genetic background. For understanding the underlying mechanisms driving these changes, further studies are required, including cross-resistance profiling for different antibiotics, biofilm inhibition assays, qPCR and mutation repair assays. Additionally, sequencing and genetic analysis of the evolved samples to identify SNPs and other mutations responsible for these tolerance capabilities.

